# Antigenic Characterization of Human Monoclonal Antibodies for Therapeutic Use against H7N9 Avian Influenza Virus

**DOI:** 10.1128/jvi.01431-22

**Published:** 2022-12-21

**Authors:** Pengxiang Chang, Deimante Lukosaityte, Joshua E. Sealy, Pramila Rijal, Jean-Remy Sadeyen, Sushant Bhat, Sylvia Crossley, Rebecca Daines, Kuan-Yin A. Huang, Alain R. Townsend, Munir Iqbal

**Affiliations:** a The Pirbright Institute, Pirbright, Woking, United Kingdom; b Center for Translational Immunology, Chinese Academy of Medical Science Oxford Institute, Nuffield Department of Medicine, University of Oxford, Oxford, United Kingdom; c MRC Human Immunology Unit, MRC Weatherall Institute of Molecular Medicine, Radcliffe Department of Medicine, University of Oxford, Oxford, United Kingdom; d Graduate Institute of Immunology, College of Medicine, National Taiwan University, Taipei, Taiwan; e Department of Pediatrics, National Taiwan University Hospital, Taipei, Taiwan; St. Jude Children’s Research Hospital

**Keywords:** avian influenza, H7N9, mutations, antigenic site residues, hemagglutination, immune escape, monoclonal antibodies, pH fusion, receptor binding, thermal stability

## Abstract

Since 2013, H7N9 avian influenza viruses (AIVs) have caused more than 1,500 human infections and the culling of millions of poultry. Despite large-scale poultry vaccination, H7N9 AIVs continue to circulate among poultry in China and pose a threat to human health. Previously, we isolated and generated four monoclonal antibodies (mAbs) derived from humans naturally infected with H7N9 AIV. Here, we investigated the hemagglutinin (HA) epitopes of H7N9 AIV targeted by these mAbs (L3A-44, K9B-122, L4A-14, and L4B-18) using immune escape studies. Our results revealed four key antigenic epitopes at HA amino acid positions 125, 133, 149, and 217. The mutant H7N9 viruses representing escape mutations containing an alanine-to-threonine substitution at residue 125 (A125T), a glycine-to-glutamic acid substitution at residue 133 (G133E), an asparagine-to-aspartic acid substitution at residue 149 (N149D), or a leucine-to-glutamine substitution at residue 217 (L217Q) showed reduced or completely abolished cross-reactivity with the mAbs, as measured by a hemagglutination inhibition (HI) assay. We further assessed the potential risk of these mutants to humans should they emerge following mAb treatment by measuring the impact of these HA mutations on virus fitness and evasion of host adaptive immunity. Here, we showed that the L4A-14 mAb had broad neutralizing capabilities, and its escape mutant N149D had reduced viral stability and human receptor binding and could be neutralized by both postinfection and antigen-induced sera. Therefore, the L4A-14 mAb could be a therapeutic candidate for H7N9 AIV infection in humans and warrants further investigation for therapeutic applications.

**IMPORTANCE** Avian influenza virus (AIV) H7N9 continues to circulate and evolve in birds, posing a credible threat to humans. Antiviral drugs have proven useful for the treatment of severe influenza infections in humans; however, concerns have been raised as antiviral-resistant mutants have emerged. Monoclonal antibodies (mAbs) have been studied for both prophylactic and therapeutic applications in infectious disease control and have demonstrated great potential. For example, mAb treatment has significantly reduced the risk of people developing severe disease with severe acute respiratory syndrome coronavirus 2 (SARS-CoV-2) infection. In addition to the protection efficiency, we should also consider the potential risk of the escape mutants generated by mAb treatment to public health by assessing their viral fitness and potential to compromise host adaptive immunity. Considering these parameters, we assessed four human mAbs derived from humans naturally infected with H7N9 AIV and showed that the mAb L4A-14 displayed potential as a therapeutic candidate.

## INTRODUCTION

Since February 2013, a novel H7N9 avian influenza virus (AIV) has caused 1,568 confirmed human infections and 616 deaths, with an ~40% case fatality rate (1). Although most human infections are linked to direct contact with birds or visiting live-poultry markets, some hospital or family infection clusters have been observed, raising the concern of possible but limited human-to-human transmission (2). Therefore, H7N9 AIV has been considered a credible pandemic threat.

During early epidemic waves, only low-pathogenicity avian influenza (LPAI) virus was detected, while the high-pathogenicity avian influenza (HPAI) H7N9 virus emerged in late 2016, causing up to 100% mortality in infected chickens (3). Given the threat of H7N9 AIV to human and animal health, the Chinese government implemented a mass vaccination program targeting poultry in 2017. As a result, the numbers of poultry outbreaks and human infections have dropped dramatically, with only three human infection cases being reported from 2016 to 2017 and one human infection case being reported from 2017 to 2018; no further human infections have been reported to date (1). However, these viruses have not been eradicated, with the continuous sporadic isolation of LPAI and HPAI H7N9 viruses in poultry (4, 5).

Neuraminidase (NA) inhibitors, such as zanamivir (Relenza) and oseltamivir (Tamiflu), are the major antivirals that have been recommended for the treatment of severe infection with AIV in humans. However, the rapid emergence of NA inhibitor-resistant H7N9 viruses highlights the need for new anti-influenza drugs or therapeutics (6), including the application of human antibodies. Li et al. isolated a monoclonal antibody (mAb) (P52E03) recognizing glycine (G) at amino acid residue 133 in hemagglutinin (HA) and demonstrated its ability to protect against lethal H7N9 AIV challenge in a mouse model (7). Additionally, mAb m826 has been shown to recognize a pH-sensitive epitope, also providing full protection in mice challenged with a lethal dose of H7N9 AIV (8). Chen et al. and Wang et al. isolated neutralizing antibodies, namely, HNIgGD5, HNIgGH8, HNIgGA6, and HNIgGB5, targeting the highly conserved epitopes valine (V) at amino acid residue 177 and leucine (L) at amino acid residue 226 (9, 10) in HA. Another mAb, H7.167, targeting the highly conserved epitope asparagine (N) at amino acid residues 146 and 149, significantly reduced viral lung titers in a mouse intranasal virus challenge study (11).

We previously identified four H7N9 human IgG antibodies, namely, L4A-14, L3A-44, K9B-122, and L4B-18, from humans naturally infected with H7N9 AIV (12). L4-A14 and L3A-14 showed robust therapeutic and prophylactic efficacy against HPAI H7N9 (A/Guangdong/TH005/2017), while L4B-18 failed to protect mice from lethal infection. As for the LPAI H7N9 virus strain A/Taiwan/1/2013, although L4A-14, L3A-44, and K9B-122 all showed robust prophylactic protection, 100% survival was found only with antibody L4A-14 in a therapeutic model (12).

In this study, we mapped H7N9 HA epitopes using a mAb escape mutant method and investigated the cross-reactivity of these H7HA mAbs with prevalent H7N9 AIVs. The emergence of virus mutants escaping neutralization following immune pressure, such as that from antibody treatment, is inevitable; therefore, it is critical to investigate the risk of these mutants to public health. Efficient human-to-human transmission requires a reduced threshold for pH stability, increased HA thermal stability, and a shift of the HA binding affinity from the avian to the human receptor (13, 14). We therefore assessed the risk of these mAb escape mutants to human health by evaluating the impact of these HA substitutions on receptor binding, the pH of fusion, thermal stability, and virus replication fitness. Moreover, we assessed the evasion of vaccine- or infection-induced humoral adaptive immunity by microneutralization (MN) and hemagglutination inhibition (HI) assays with homologous postinfection or postvaccination sera.

## RESULTS

### Biological characterization of human mAbs against H7N9 AIV.

**FIG 1:**
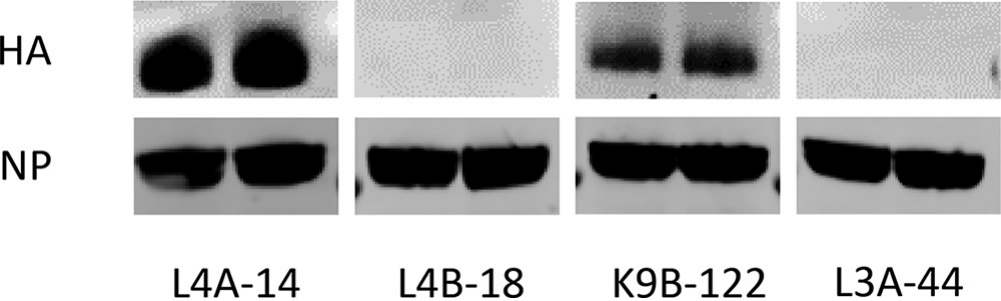
Western blotting to detect HA by human anti-H7N9 HA monoclonal antibodies. The HA from H7N9 influenza virus (A/Anhui/1/2013) was probed with human monoclonal antibody L4A-14, L4B-18, K9B-122, or L3A-44 with nucleoprotein (NP) detection as a loading control. Three microliters of purified H7N9 Anhui/13 viruses (virus concentration of 10 μM) was lysed and loaded per sample, with two replicates for each antibody.

Four recombinant mAbs, L4A-14, K9B-122, L3A-44, and L4B-18, were produced in ExpiCHO-S cells and purified by protein G affinity chromatography. To assess the neutralizing profiles of the mAbs, the HI and MN titers were assessed by using reassortant H7N9 (A/Anhui/2013 [referred to as Anhui/13 here]) with HA and NA from H7N9 AIV and the six internal genes from PR8 (A/Puerto Rico/8/34 [H1N1]) virus. All four mAbs showed HI activity against H7N9 AIV, with stronger HI activity being observed with L4A-14, followed by L3A-44 and K9B-122 and L4B-18 showing relatively lower HI titers (Table 1). The MN titers followed a trend similar to that of the HI titers, but no detectable MN activity was found with L4B-18. The mAbs were used to detect the H7N9 HA by Western blotting by denaturing gel electrophoresis. L4A-14 and K9B-122 recognized the H7N9 HA under denaturing conditions, whereas no H7 HA antigen band was detected with the L3A-44 and L4B-18 mAbs (Fig. 1).

**TABLE 1:** Properties of the human anti-H7N9 monoclonal antibodies^*a*^

mAb	HI titer	MN titer	Western blotting result
L4A-14	4,096	224	+
K9B-122	1,024	96	+
L3A-44	2,731	149	−
L4B-18	640	ND	−

a mAb, monoclonal antibody; HI, hemagglutination inhibition; MN, microneutralization; ND, not detected. “+” indicates that Western blotting was positive; “−” indicates that Western blotting was negative. The HI titers and MN titers were normalized for antibody at a concentration of 1 mg/mL given the different starting concentrations of antibodies.

### H7N9 HA epitope mapping by selection of mAb escape mutants.

To select the mAb escape mutants, an H7N9 Anhui/13 AIV stock was 10-fold serially diluted and then incubated with 1 mg/mL of each mAb, and the antibody-virus mixture was then inoculated into 10-day-old embryonated eggs. The HA-positive allantoic fluid from the embryonated eggs inoculated with the lowest concentration of virus was selected for viral HA gene sequence analysis.

**FIG 2:**
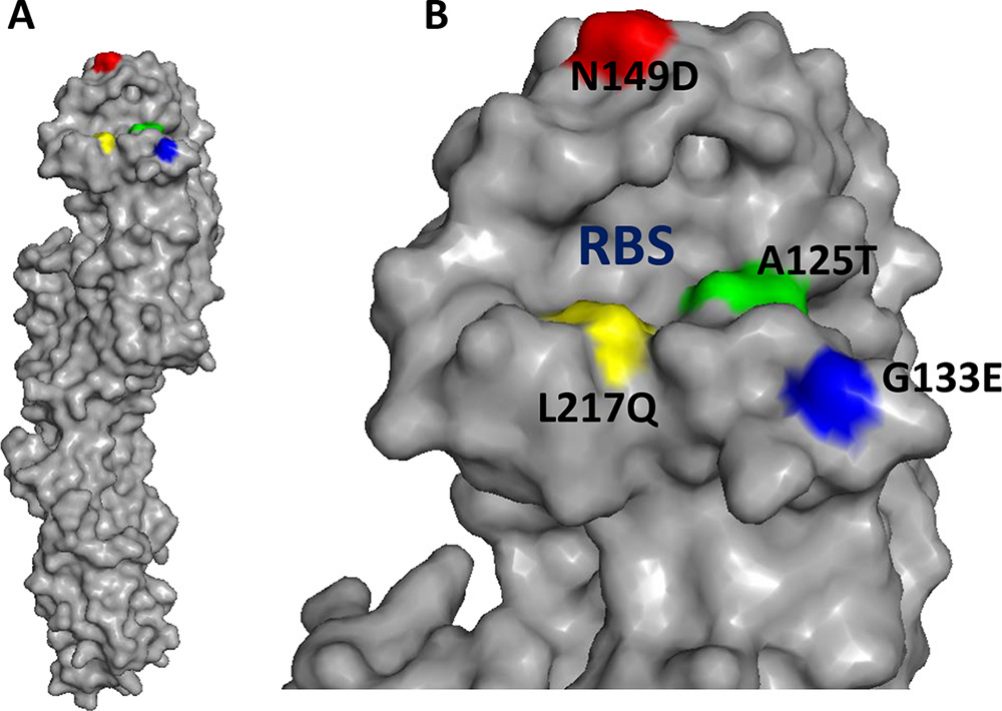
Location of the HA mutation residues on the HA monomer (A) and HA1 head domain (B) of the three-dimensional structure of H7N9 influenza virus (A/Shanghai/1/2013) (Protein Data Bank [PDB] accession number 4LN3). RBS, receptor binding site. The mutations A125T, G133E, N149D, and L217Q (mature H7 HA numbering) are indicated in green, blue, red, and yellow, respectively.

The results showed that the propagation of virus in the presence of the monoclonal antibodies gave rise to the following substitutions: N to D at residue 149 (N149D) for L4A-14, G to E at residue 133 (G133E) for K9B-122, A to T at residue 125 (A125T) for L3A-44, and L to Q at residue 217 (L217Q) for L4B-18 (mature H7 numbering, which is used throughout, corresponding to N158D, G144E, and A135T, and L226Q, respectively, by H3 numbering). These substitutions are positioned within the respective footprints of the antibodies that selected them (12). In addition, the A125T substitution targeted by L3A-44 resulted in the formation of an N-linked glycan at amino acid residue 123 (15), which is also within the footprint of antibody L3A-44 (12). N149D is located at the top of the HA, and both the A125T and L217Q mutations are located close to the receptor binding site (RBS), while G133E is located at the edge of the RBS (Fig. 2).

To confirm the functional role of these substitutions in the escape mutants, each of the identified mutations was engineered into the wild-type HA of Anhui/13 reverse genetic (RG) virus that contained the six internal genes of the PR8 virus. The HI assay showed that the mutant virus with HA substitutions G133E and A125T completely lost HI activity with mAb K9B-122 and L3A-44, respectively (Table 2). The L217Q mutant virus showed a 32-fold HI titer reduction for the L4B-18 mAb, while the N149D mutant virus showed a 4-fold HI titer reduction with L4A-14, compared with the parent virus.

**TABLE 2:** Amino acid substitutions identified in the HAs of the human monoclonal antibody escape mutants

Selection mAb	Mutation	Fold reduction in HI titer by escape mutant
L4A-14	N149D	4
K9B-122	G133E	1,024
L3A-44	A125T	4,096
L4B-18	L217Q	32

### Cross-reactivity of H7N9 AIV escape mutants with the mAbs.

To investigate the cross-reactivity of the H7N9 HA mutants with the individual mAbs, all of the selected escape mutants were tested by an HI assay with the panel of mAbs. Additionally, we included mutant A151T because it introduced an N-linked glycosylation site at amino acid residue 149, a regular strategy for influenza viruses to escape neutralizing antibodies by attaching glycans, which can sterically block the binding of antibodies to multiple antigenic sites (12, 15, 16). We also included the A125V mutant since 95% of the H7N9 AIV isolates in the recent epidemic wave (wave 5) contain this mutation in HA (17). L4A-14 exhibited broad neutralizing activity against almost all of the mutants tested, except for the A151T substitution in HA, which completely abolished its HI activity (Table 3). Interestingly, under L4A-14 mAb selection pressure, only the N149D mutant emerged, rather than the A151T mutant, with only a 4-fold reduction in the HI titer. L3A-44 showed reduced cross-reactivity against the G133E mutant; only a slight HI titer reduction was observed with the A125V and L217Q mutants. L4B-18 showed a moderate reduction in cross-reactivity with the A151T, A125T, and A125V mutants while maintaining its cross-reactivity with the N149D and G133E mutants. Among these antibodies, K9B-122 was most sensitive to mutations, completely losing cross-reactivity with the A125V, A125T, L217Q, and G133E mutants apart from the N149D and A151T mutants.

**TABLE 3:** Cross-reactivity of H7N9 HA mutants with H7HA mAbs^*a*^

Selection mAb	Mutation	Fold reduction in HI titer by escape mutant			
		L4A-14	L3A-44	L4B-18	K9B-122
L4A-14	N149D	4	2	0	0
	A151T	4,096	2	4	0

L3A-44	A125T	0	4,096	8	1,024
	A125V	0	4	4	1,024

L4B-18	L217Q	2	4	32	1,024

K9B-122	G133E	0	512	0	1,024

a The A151T mutant was additionally included in this analysis because it introduced an N-linked glycosylation site at amino acid residue 149 in H7N9 HA; mutant A125V was additionally included since 95% of the H7N9 AIV isolates in the recent epidemic wave (wave 5) contain this mutation in HA.

### Cross-reactivity of recent H7N9 HA AIVs with the mAbs.

To investigate the cross-reactivity of the mAbs against recent H7N9 AIV isolates, we performed HI assays against both LPAI (A/Hong Kong/125/2017 [HK125]) and HPAI (A/Guangdong/17SF003/2016 [GDSF003]) viruses from epidemic wave 5. Additionally, we included reassortant Anhui/13 with A125T, A151T, and L217Q substitutions in HA, as these three mutations have been found in recent H7N9 isolates from poultry in China as well as H7N9 virus that is serially passaged in the presence of homologous ferret serum (5, 17). The A125T A151T L217Q mutant completely lost its cross-reactivity with all of the mAbs, except for low cross-reactivity to L4B-18 (Table 4). mAb L4A-14 showed strong cross-reactivity with both the HPAI GDSF003 and LPAI HK125 viruses, while mAb K9B-122 lost cross-reactivity with all of the H7N9 AIVs tested, apart from Anhui/13, which confirmed previous results obtained by pseudotype neutralization (12). The mAbs L3A-44 and L4B-18 still maintained moderate cross-reactivity with HK125; however, significant reductions in the HI titers, 256-fold and 128-fold, respectively, were observed with GDSF003. These results suggest that mAb L4A-14 harbors robust neutralizing potential with isolates from epidemic wave 5, which presented the highest rate of human infection with H7N9 AIV. However, the A151T substitution would inevitably render viruses resistant.

**TABLE 4:** Cross-reactivity of mAbs with recent H7N9 AIVs^*a*^

mAb	HI titer			
	Anhui/13	GDSF003	HK125	A125T A151T L217Q
L4A-14	4,096	1,024	4,096	0
L3A-44	4,096	8	256	0
L4B-18	512	4	128	2
K9B-122	1,024	0	0	0

a Anhui/13, reassortant H7N9 A/Anhui/2013; HK125, reassortant H7N9 A/Hong Kong/125/2017; GDSF003, reassortant H7N9 A/Guangdong/17SF003/2016; A125T A151T L217Q, reassortant Anhui/13 with HA mutations A125T, A151T, and L217Q.

### Cross-reactivity of H7N9 AIV escape mutants with homologous sera.

**FIG 3:**
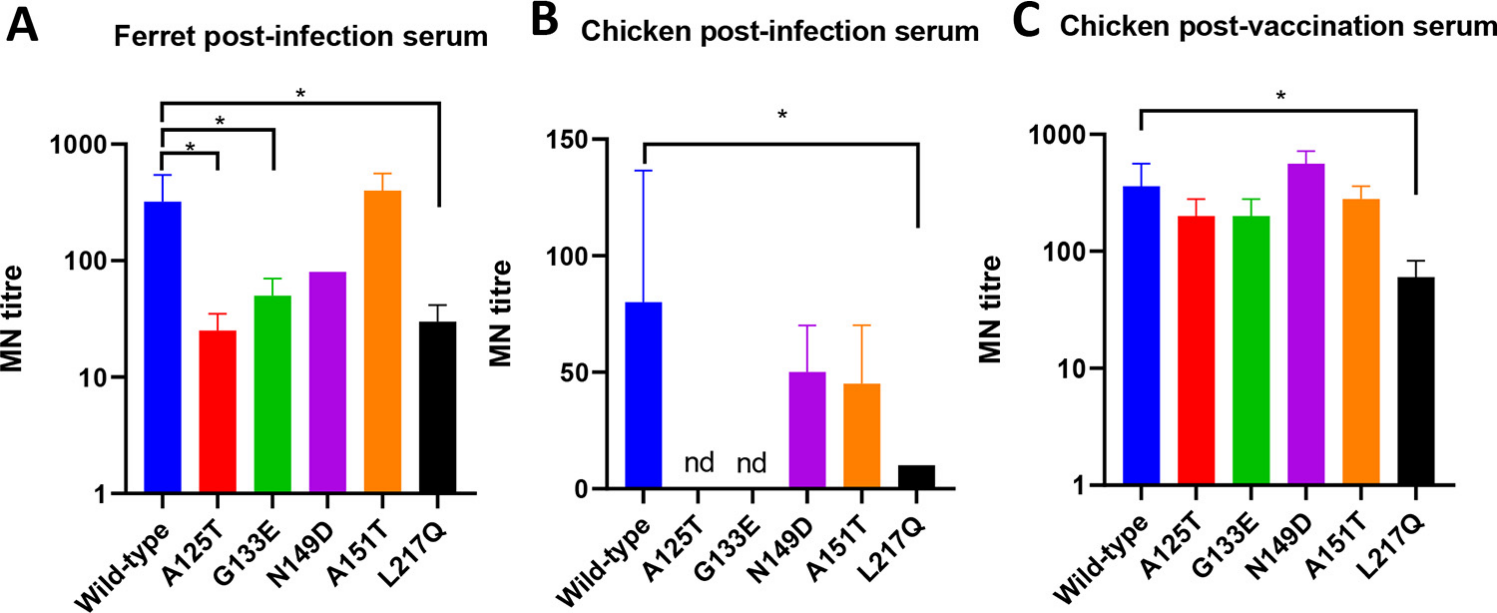
Neutralization by ferret postinfection serum (A), chicken postinfection serum (B), and chicken postvaccination serum (C) against wild-type Anhui/13 and its mAb escape mutants. The A151T mutant was included since it resulted in glycosylation at amino acid residue 149 and completely lost cross-reactivity with mAb L4A-14. Error bars indicate standard errors of the means. *, *P* < 0.05. nd, no detection; MN, microneutralization.

It is inevitable that escape mutants will emerge following the therapeutic use of mAbs. Therefore, it is critical to assess the risk of these mutants to public health before their application as therapeutics for human H7N9 AIV infection. Ideally, the escape mutant should not be able to evade infection- or vaccine-induced immunity. To this end, we checked the HI and MN titers of the mAb escape mutants with both chicken and ferret postinfection sera. Additionally, we included chicken antiserum raised against inactivated H7N9 virus to mimic chicken postvaccination serum. In agreement with the results of a previous study, L217Q resulted in a significant drop in the HI titer against both ferret postinfection serum (16-fold) and chicken postvaccination serum (8-fold) (Table 5) (17). However, only a 2-fold change in the HI titer was observed with chicken postinfection serum. The A125T and A151T substitutions in HA resulted in slight reductions in the HI titers against both ferret postinfection serum and chicken postvaccination serum, whereas no change was observed with chicken postinfection serum. The G133E mutant showed HI titers comparable to those of the wild type against both chicken postinfection and postvaccination sera. Interestingly, a 2-fold increase in the HI titer was observed with ferret postinfection serum. The N149D substitution in HA had no obvious impact on the HI titer against chicken vaccination serum, but surprisingly, a 2-fold increase in the titer was observed with both ferret and chicken postinfection sera. Consistent with the results of the HI assay, all of the sera tested showed reduced MN titers against L217Q, while no significant changes were observed with the N149D and A151T mutant viruses (Fig. 3A to C). In contrast to the HI assay results, the ferret and chicken postinfection sera showed significant reductions in the MN titers against the A125T and G133E mutants, while no significant change was observed with chicken postvaccination serum.

**TABLE 5:** Cross-reactivity of H7N9 AIV escape mutants with homologous sera

HA mutation	HI titer		
	Ferret serum (postinfection)	Chicken serum (postinfection)	Chicken serum (postvaccination)
Wild type	1,024	320	512
A125T	256	320	256
G133E	2,048	320	512
N149D	2,048	640	512
A151T	512	320	256
L217Q	64	160	64

To conclude, the A125T, G133E, and L217Q substitutions in HA resulted in significant decreases in cross-reactivity to both chicken and ferret postinfection sera; however, only the L217Q mutant showed reduced cross-reactivity against chicken postvaccination serum compared to wild-type Anhui/13. All of the postinfection and postvaccination sera tested here demonstrated comparable neutralizing profiles among wild-type Anhui/13, mAb L4A-14 escape mutant N149D, and mutant A151T, having completely lost HI activity against mAb L4A-14.

### The A125T and N149D mutants replicated robustly *in vitro* and *in ovo*.

To evaluate the effects of mAb escape mutations in the HA on virus replication fitness, we generated a series of reverse-genetics-based mutant viruses carrying the escape mutations A125T, G133E, N149D, and L217Q by site-directed mutagenesis. We did not include the A151T mutant for further analysis because it did not emerge under mAb pressure. The mutant with A151T substitution in HA also showed a significant drop in viral thermal stability as well as reduced receptor binding to human-like receptors. Therefore, it is unlikely to pose an increase zoonotic risk (15).

**FIG 4:**
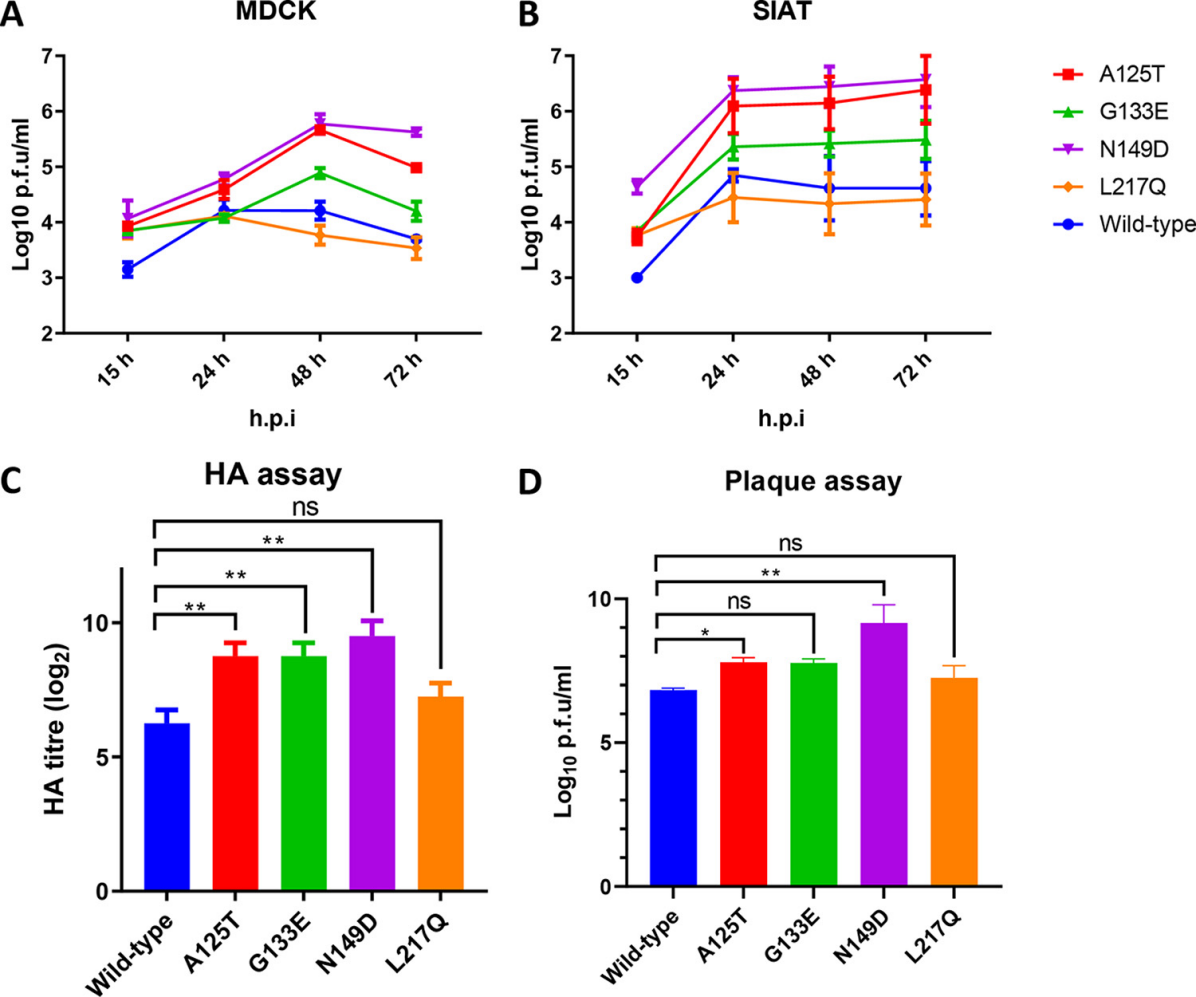
Replication of reassortant Anhui/13 and its mAb escape mutants *in ovo* and in MDCK and SIAT cells. (A and B) Replication kinetics of wild-type Anhui/13 and its mutants in MDCK cells (A) and SIAT cells (MDCK cells modified to express a higher density of α2,6 human receptors) (B). Cells were infected at a multiplicity of infection (MOI) of 0.001; the supernatants were collected at 15, 24, 48, and 72 h postinfection (h.p.i); and the virus titers were determined by plaque assays in MDCK cells (for the results of statistical analysis, see Tables S1 and S2 in the supplemental material). (C and D) Ten-day-old embryonated eggs were inoculated with 100 PFU of wild-type Anhui/13 and its mutants for 48 h before allantoic fluid was harvested and viral replication was determined by an HA assay (C) and a plaque assay (D). Error bars indicate standard errors of the means. *, *P* < 0.05; **, *P* < 0.001; ns, not significant.

We assessed the propagation for each of the viruses in mammalian Madin-Darby canine kidney (MDCK) cells and MDCK-SIAT1 (SIAT) cells, which have been modified to express a higher density of α2,6-sialyltransferase (18). Similar to the wild type, the L217Q mutant replicated poorly in MDCK cells despite higher titers at 15 h postinfection (hpi) (Fig. 4A). The A125T, G133E, and N149D mutants replicated significantly better than the wild type in MDCK cells at all of the time points except 24 hpi, where only mutant N149D replicated to a significantly higher titer than that of the wild type (Fig. 4A). As for the SIAT cells, both the A125T and N149D mutants replicated to significantly higher titers than those of the wild type at 24, 48, and 72 hpi, with only N149D having a significantly higher titer than that of the wild type at 15 hpi (Fig. 4B). Both the L217Q and G133E mutants replicated comparably to the wild type, although the titer of the G133E mutant was slightly higher than that of the wild type throughout. To further assess the effects of these mutations on virus replication, 10-day-old embryonated eggs were inoculated with 100 PFU of wild-type Anhui/13 and its mutants for 48 h, and virus titers were determined by an HA assay (Fig. 4C) and a plaque assay (Fig. 4D). The replication of the L217Q mutant was comparable to that of wild-type Anhui/13, while all of the other mutants showed significantly higher replication titers. The plaque assay results showed patterns similar to those for the HA assay except that the replication titer for the G133E mutant was not statistically different from that of the wild-type Anhui/13 virus (Fig. 4C and D). To conclude, the A125T and N149D mAb escape mutants demonstrated enhanced replication fitness both *in vitro* and *in ovo*.

### mAb escape mutations affected H7N9 AIV receptor binding properties.

**FIG 5:**
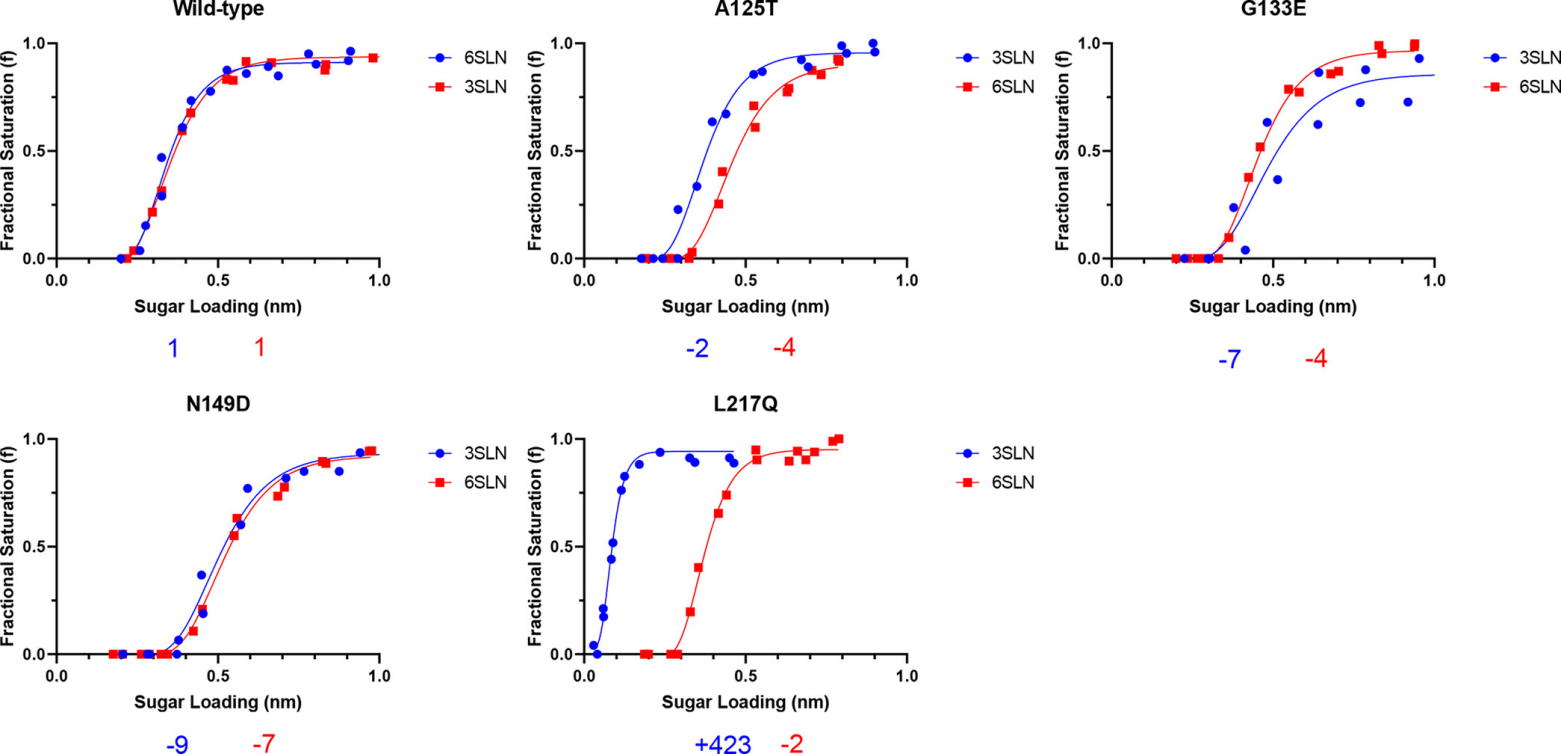
Receptor binding profiles of reassortant Anhui/13 and its mAb escape mutants. The binding of purified reassortant Anhui/13 (wild-type) virus and the A125T, G133E, N149D, and L217Q mutants to avian (3SLN) and human (6SLN) receptor analogues was determined by biolayer interferometry. The numbers below each figure show the fold changes in the receptor binding of the indicated viruses compared to the reassortant Anhui/13 (wild-type) virus. − indicates a reduction, and + indicates an increase. Data are the combination of results from two repeats for each virus-receptor analogue combination.

Receptor binding affinity is one of the key determinants of the interspecies transmission of influenza viruses. To examine the impacts of mAb escape mutations on virus receptor binding properties, we utilized biolayer interferometry to characterize the receptor binding of these viruses to the avian-like and human-like receptor analogues 3′-sialylacetyllactosamine (3SLN) and 6′-sialylacetyllactosamine (6SLN). In agreement with previous reports, H7N9 Anhui/13 showed comparable binding to both the 3SLN and 6SLN receptor analogues, and the L217Q mutant showed a considerable increase in binding to avian 3SLN (~432-fold), with only a slight decrease (~2-fold) in binding to 6SLN analogues (Fig. 5) (15, 19). Substitutions A125T, G133E, and N149D reduced receptor binding to both the 3SLN and 6SLN analogues. Compared with the A125T mutant (~2-fold reduction in binding to 3SLN and ~4-fold reduction in binding to 6SLN), there was a slightly greater reduction with mutant G133E (~7-fold reduction in binding to 3SLN and ~4-fold reduction in binding to 6SLN) and mutant N149D (~9-fold reduction in binding to 3SLN and ~7-fold reduction in binding to 6SLN).

### mAb escape mutations affected H7N9 AIV pH of fusion and thermal stability.

**FIG 6:**
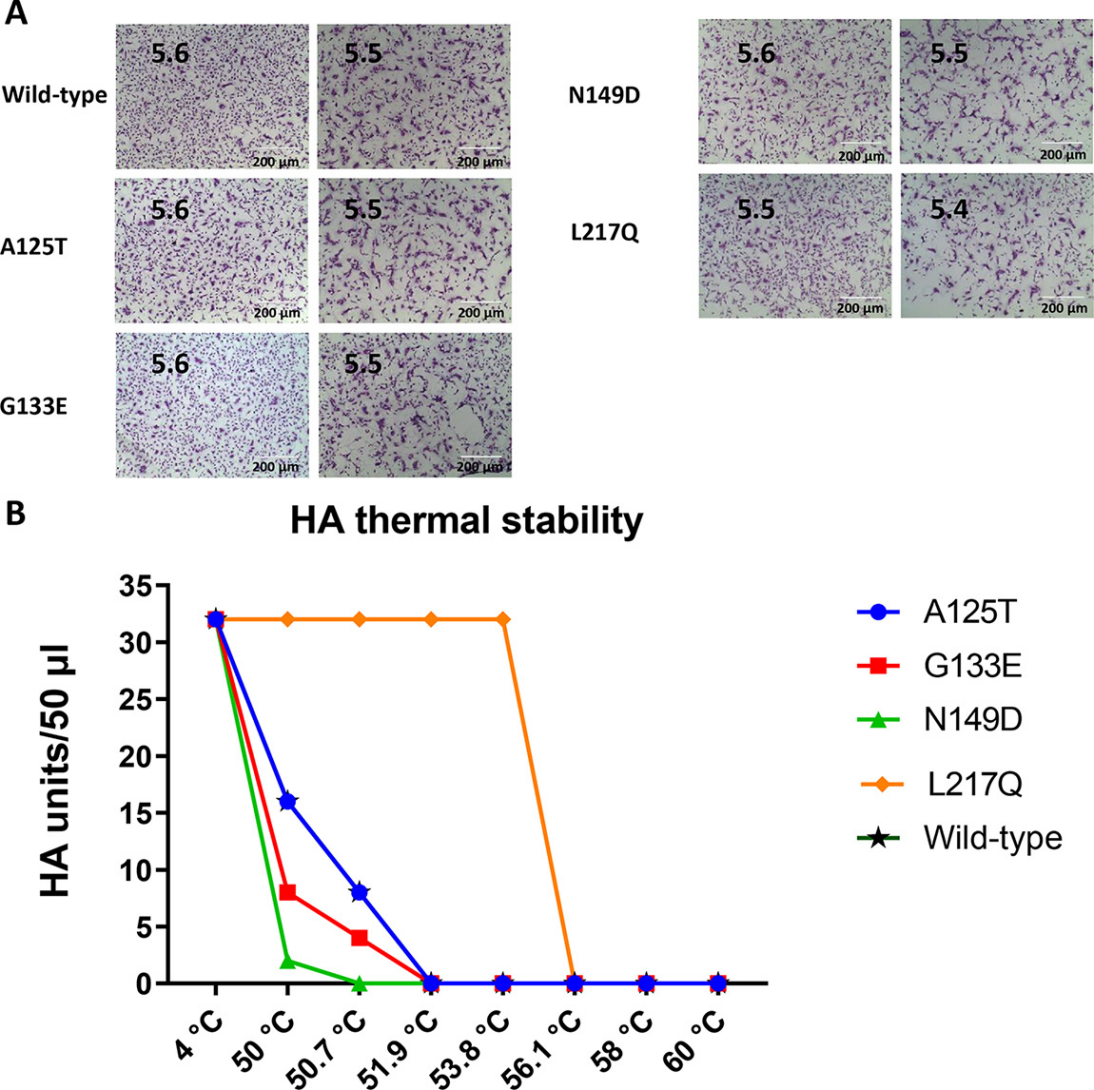
pH of fusion and thermal stability of reassortant Anhui/13 and its mAb escape mutants. (A) Syncytium formation in Vero cells infected with the reassortant Anhui/13 (wild-type) virus and the A125T, G133E, N149D, and L217Q mutants. The pH at which 50% maximum syncytium formation was observed was taken as the predicted pH of fusion, shown on the left. Syncytium formation at 0.1 pH units lower than the fusion threshold is shown on the right as a control. The results shown are representative of data from three experimental repeats. (B) HA thermal stability of reassortant wild-type Anhui/13 and the A125T, G133E, N149D, and L217Q mutants, with wild-type Anhui/13 as a control. Thirty-two hemagglutinating units of reassortant virus were either left in a 4°C fridge as a control or heated at 50°C, 50.7°C, 51.9°C, 53.8°C, 56.1°C, 58.0°C, 59.2°C, and 60°C for 30 min before the HA assay. The results shown are representative of data from three experimental repeats.

The pH of fusion has been shown to play an important role in host adaptation and transmission (20). Consistent with a previous report, wild-type Anhui/13 triggered fusion at pH 5.6 or lower (Fig. 6A) (21). The A125T, G133E, and N149D mAb escape mutants showed pH fusion thresholds similar to that of wild-type Anhui/13. Only the L217Q substitution in HA caused a slight decrease in the pH fusion threshold.

In addition to receptor binding and the pH of fusion, HA thermal stability also plays a vital role in AIV evolution (13, 14). Wild-type Anhui/13 and the mAb escape mutants were heated at 50°C, 50.7°C, 51.9°C, 53.8°C, 56.1°C, 58.0°C, 59.2°C, and 60°C for 30 min, after which the loss of HA activity was determined by an HA assay. The G133E and A125T mutants showed heat stability profiles similar to that of wild-type Anhui/13. The L217Q mutation dramatically increased HA stability, while the N149D mutation slightly reduced HA stability (Fig. 6B).

## DISCUSSION

In our previous study, we isolated four potent monoclonal antibodies from humans naturally infected with H7N9 AIV. To gain in-depth knowledge of the therapeutic potential of these antibodies, we mapped the antigenic epitopes of these antibodies by escape mutant selection methods. In addition to the neutralizing capabilities, we also evaluated the potential risk to humans of mAb-induced escape mutants by assessing their replication fitness, receptor binding, pH of fusion, thermal stability, and potential to evade host adaptive immunity. Overall, the L4A-14 mAb showed robust neutralizing capabilities and broad cross-reactivity to H7N9 AIVs. Moreover, its escape mutant N149D demonstrated reduced thermal stability while retaining strong antigenic cross-reactivity with both postinfection and postvaccination sera, making the L4A-14 mAb a suitable therapeutic candidate for further investigation.

The amino acid substitutions selected by the three human mAbs for which we have crystal structures, L4A-14, L3A-44, and L4B-18, can be interpreted logically by reference to the antibody footprints defined by crystallography (12). L4A-14 selects for N149D and is also blocked by A151T that introduces an N-linked glycosylation site at N149. N149 is situated close to the sialic acid binding site and is contacted by the light chain of the antibody. L3A-44 selects for A125T. A125 is also close to the sialic acid binding site, and the introduction of threonine at that position would introduce an N-linked glycosylation site at N123, which is also contacted by the heavy chain of antibody L3A-44. L4B-18 selected for substitution L217Q. This is a naturally occurring substitution that appeared in H7N9 AIVs isolated in 2016 and beyond (A/Guangdong/17SF003/2016). L217 is also close to the sialic acid binding site and is contacted by the heavy chain of L4B-18. We note that this antibody has selected exactly residue Q217 that occurs in nature. This underlines the capacity for selection by human mAbs *in vitro* to predict the evolution of influenza viruses *in vivo*, as seen for human mAbs to seasonal H1N1 (22). In that example, human mAbs selected for a lysine-to-glutamine substitution at amino acid 163 in HA that became dominant in viruses isolated after 2013.

H7N9 AIV is still circulating among poultry in China, frequently causing human infection. Fortunately, few cases of human infection have occurred since the Chinese government introduced large-scale poultry vaccinations. We previously used an *in vitro* immune escape mutant selection method to model H7N9 evolution under immune pressure using ferret H7N9 antisera (17). We predicted that the mutations A125T, A151T, and L217Q in HA might occur under the pressure of humoral immunity, all of which have been found in recent strains of H7N9 AIV isolated from poultry in China (5). Here, we showed that three mAbs isolated from humans naturally infected with H7N9 also target HA amino acid positions 125, 149, and 217. The consistency between these two studies indicated that these epitopes are the dominant epitopes and that ferrets might have antibody response profiles very similar to those of humans, further supporting the ferret model for human candidate vaccine selection. We found that the A125T A151T L217Q mutant lost cross-reactivity with almost all mAbs, maintaining only limited cross-reactivity with the L4B-18 mAb, implying that these mAbs may provide little protection against H7N9 AIVs containing such mutations. However, it is noteworthy that these mutations, particularly A125T and A151T double mutations in HA, resulted in the complete loss of human receptor binding and decreased HA thermal stability, making them less likely to emerge in an H7N9 AIV pandemic (15). This is further supported by the finding that only one human infection case of H7N9 AIV contained both the A125T and A151T mutations in the HA (23). Although the A125T and A151T mutations resulted in the complete loss of cross-reactivity with the L3A-44 and L4A-14 mAbs, respectively, the L4A-14 mAb retained antigenic cross-reactivity against L3A-44 mAb escape mutants and vice versa. Therefore, antibody treatment containing a combination of the L3A-44 and L4A-14 mAbs could be a highly efficient therapeutic combination for neutralizing viruses containing A125T or A151T substitutions.

Interestingly, L4A-14 induced the escape mutant N149D rather than the A151T mutant, with significant antigenic change. One possible explanation is that A151T substitutions result in considerable reductions in HA stability and receptor binding, negatively impacting viral fitness, compared to mutant N149D (15).

The Q217L substitution in HA has been linked to a switch in the binding affinity from avian receptors to human receptors (α2,3 to α2,6) in H2, H3, and H4 avian influenza viruses (24–26), which could be a prerequisite for zoonotic infection. The L217Q substitution has been shown to hinder the transmission of H7N9 AIV between pigs (27). Similarly, Sun et al. reported that LPAI H7N9 virus containing L217 was transmitted well between ferrets, while two HPAI H7N9 viruses containing Q217 was not (28). Here, we showed that L217Q mutants emerged under L4B-18 mAb pressure *in ovo*. Further investigation is required to justify the application of this mAb for passive immunization *in vivo*, with the aim to drive the virus mutation L217Q with lower pandemic potential.

We previously showed that the amino acid at position 217 is a key mediator of H7N9 AIV antigenicity. Both HI and MN assay results support this finding, demonstrating that the L217Q substitution in HA resulted in a significant reduction in antigenic cross-reactivity with both ferret and chicken postinfection sera as well as chicken postvaccination serum. In contrast, Wang et al. concluded that L217Q had a minimal impact on the antigenicity of H7N9 AIV (29); however, this discrepancy could be due to the different animal models used, mouse and macaque, and we agree that multiple serological assays should be taken into consideration when evaluating antigenic variation. Although no HI titer change was observed with the A125T mutant, a considerable reduction in the MN titer was detected with chicken postinfection serum. Similarly, despite comparable HI titers, the G133E mutant showed significant decreases in MN titers against both chicken and ferret postinfection sera. Interestingly, we found that A125T, G133E, and L217Q caused significant antigenic change in postinfection sera of both chickens and ferrets. However, only substitution L217Q resulted in significant antigenic changes in the postvaccination sera of both hosts. It appeared that the postvaccination serum from chickens vaccinated with adjuvanted inactivated virus is more broadly cross-reactive than the postinfection serum. This difference could be due to the effects of the adjuvant, which has been shown to affect virus antigenicity (30). Unfortunately, the chicken postvaccination and postinfection sera were not produced in the same experiment with proper control, thus warranting further investigation to understand the conflicting phenomena found in this study.

To conclude, we evaluated the potential of utilizing human-derived mAbs raised against H7N9 AIV as an infection therapy. However, immune pressure, including mAb therapy, will inevitably induce escape mutations. As such, we identified and mapped the H7N9 HA by immune escape *in ovo*. Additionally, we assessed the potential zoonotic risk and the viral fitness consequences of the identified mutations. Our data revealed that the L4A-14 mAb could be a potential therapeutic candidate for human H7N9 infections, with escape mutants posing a lower risk to human health and, therefore, lower pandemic potential.

## MATERIALS AND METHODS

### Ethics statement.

The embryonated chicken egg work was carried out in accordance with the guidance and regulations of the United Kingdom Home Office under project license number P68D44CF4. All of the influenza virus-related work was carried out under biosafety level 2 conditions.

### Viruses and cells.

The reassortant H7N9 viruses containing HA and neuraminidase (NA) from low-pathogenicity avian influenza (LPAI) H7N9 (A/Anhui/1/2013) virus (referred to as wild-type Anhui/13) and the internal gene segments from A/Puerto Rico/8/34 (H1N1) (PR8) were generated as previously described (17). The reassortant H7N9 AIV A125T, A151T, and L217Q mutants of Anhui/13 were generated via site-directed mutagenesis using primers described in a previous study (17). The primers for the site-directed mutagenesis of G133E and N149D are listed in Table 6. Rescued viruses were propagated in 10-day-old embryonated chicken eggs, and virus stocks were kept at −80°C.

**TABLE 6:** List of primers for H7N9 HA amplification and site-specific mutations

Primer name	Sequence (5′–3′)^*a*^
G133E of HA (Anhui/13)-Forward	GCATGTAGGAGATCAGAATCTTCATTCTATGCAG
G133E of HA (Anhui/13)-Reverse	CTGCATAGAATGAAGATTCTGATCTCCTACATGC
N149D of HA (Anhui/13)-Forward	CTGTCAAACACAGATGATGCTGCATTCCCG
N149D of HA (Anhui/13)-Reverse	CGGGAATGCAGCATCATCTGTGTTTGACAG

a The sequence for the specific amino acid mutations is underlined.

Madin-Darby canine kidney (MDCK) cells, MDCK-SIAT1 (SIAT) cells, human embryonic kidney HEK293T cells, and Vero cells (ATCC) were maintained with Dulbecco’s modified Eagle’s medium (DMEM) (Gibco) supplemented with 10% fetal calf serum (FCS) (Gibco) and 100 U/mL penicillin-streptomycin (Gibco) at 37°C under a 5% CO_2_ atmosphere.

### Monoclonal antibodies and sera.

The monoclonal antibodies were produced in ExpiCHO-S cells and purified by protein affinity chromatography. Basically, the ExpiCHO-S cells were cotransfected with antibody light and heavy chain plasmids using an ExpiFectamine CHO transfection kit according to the manufacturer’s protocol. The supernatants were filtered and purified using HiTrap protein G HP antibody purification columns (GE Healthcare).

Postinfection ferret antiserum raised against LPAI H7N9 (A/Anhui/1/2013) virus was kindly provided by John McCauley (The Francis Crick Institute, UK). The postinfection chicken antiserum was raised previously in our laboratory by infection of specific-pathogen-free (SPF)-derived Rhode Island Red chickens (Roslin Institute, UK) with reassortant LPAI H7N9 (A/Anhui/1/2013) virus (31). The sera were heat inactivated at 56°C for 30 min before further analysis.

### Replication kinetics in MDCK and MDCK-SIAT1 cells.

MDCK or MDCK-SIAT1 cells were infected with H7N9 AIV at a multiplicity of infection (MOI) of 0.001 and incubated at 37°C for 1 h. The cells were then washed once with phosphate-buffered saline (PBS) (Central Services Unit at The Pirbright Institute) and replenished with DMEM containing 2 μg/mL tosylsulfonyl phenylalanyl chloromethyl ketone (TPCK)-treated trypsin. The supernatants were taken at 15, 24, 48, and 72 h postinfection and stored at −80°C before titration by a plaque assay in MDCK cells. The plaque assay was carried out as previously described (32). MDCK cells in a 12-well plate were infected with H7N9 AIV for 1 h before the infection medium was removed. Cells were then washed once with PBS before the addition of an agarose overlay. The cells were further incubated at 37°C for 72 h before being stained with 1% crystal violet (Sigma-Aldrich).

### *In ovo* growth.

Ten-day-old embryonated chicken eggs were infected with 100 PFU of the indicated reassortant H7N9 AIV. The eggs were incubated at 35°C for 48 h before allantoic fluid was harvested for hemagglutination (HA) assays and plaque assays.

### HA assay.

The HA titration assays were performed using 1% chicken red blood cells (RBCs) as described in the WHO animal influenza training manual (33). Briefly, 50 μL of virus was 2-fold serially diluted and added to 96-well V-bottom plates (Greiner), followed by the addition of 50 μL of 1% chicken RBCs. The plates were incubated for 30 min at room temperature before the HA titer was recorded and presented as HA units per 50 μL.

### Hemagglutination inhibition assay.

Twenty-five microliters of human mAbs was 2-fold serially diluted in 96-well V-bottom plates (Greiner) with PBS prior to being mixed with an equal volume of H7N9 AIVs (8 HA units). The concentrations of mAbs L4A-14, K9B-122, L3A-44, and L4B-18 were 1.0 mg/mL, 1.5 mg/mL, 0.8 mg/mL, and 1.0 mg/mL, respectively. The antibody-virus mixtures were incubated for 1 h at room temperature and then mixed with 50 μL of 1% chicken RBCs. The plates were incubated for a further 30 min at room temperature before the hemagglutination inhibition (HI) titer was recorded as the reciprocal of the dilution that completely inhibited hemagglutination. For the data in Table 1, the HI titer was normalized as the reciprocal of the dilution of the antibody at a concentration of 1 mg/mL given the different starting concentrations of the antibodies.

### HA thermal stability assay.

The reassortant H7N9 AIVs were diluted with embryonated chicken egg allantoic fluid to 32 HA units/50 μL. The viruses were then left at 4°C as a control or heated at 50°C, 50.7°C, 51.9°C, 53.8°C, 56.1°C, 58.0°C, 59.2°C, and 60°C using a PCR thermal cycler (Bio-Rad) for 30 min before HA assays.

### Syncytium formation assay.

The pH of fusion for H7N9 AIV was determined by syncytium formation assays as previously described (34). Briefly, Vero cells in a 96-well plate were infected with H7N9 AIV, and the inoculum was aspirated at 1 h postinfection and washed once with PBS before the addition of DMEM with 10% FCS. At 16 h postinfection, cells were washed once with PBS and treated with DMEM containing 3 μg/mL TPCK-treated trypsin for 15 min. The cells were then exposed to PBS buffer at pH values ranging from 5.2 to 6.0 (in 0.1-unit increments) for 5 min. The PBS buffer was then replaced with DMEM with 10% FCS, and the cells were further incubated for 3 h at 37°C before being fixed with a methanol-acetone (1:1, by volume) mixture and stained with 20% Giemsa stain (Sigma-Aldrich) for 1 h at room temperature. The pH at which 50% maximum syncytium formation was observed was taken as the predicted pH of fusion. Images were taken on the Evos XL cell imaging system (Life Technologies).

### Virus microneutralization assay.

Monoclonal antibodies (the concentrations of mAbs L4A-14, K9B-122, L3A-44, and L4B-18 were 1.0 mg/mL, 1.5 mg/mL, 0.8 mg/mL, and 1.0 mg/mL, respectively) or polyclonal antisera were 2-fold serially diluted and then mixed with an equal volume of 100 PFU of reassortant H7N9 AIV in serum-free medium. MDCK cells were washed once with PBS before infection with the virus-serum or virus-antibody mixture for 1 h at 37°C, and the cells were then washed once with PBS and replenished with DMEM containing 2 μg/mL TPCK-treated trypsin. The cells were further kept at 37°C for 72 h before crystal violet staining. Virus microneutralization (MN) titers were expressed as the reciprocal of the highest dilution of antisera/antibodies that blocked virus infectivity in MDCK cells. The MN titers are normalized to 1 mg/mL in Table 1 given the different starting concentrations of the antibodies.

### Western blotting.

Three microliters of the sucrose-gradient-purified viruses (virus concentration of 10 μM, which was determined using an enzyme-linked immunosorbent assay against the virus NP as described previously [35]) was mixed with NuPAGE LDS sample buffer (4×) and NuPAGE sample reducing agent (10×) and heated at 70°C for 10 min before being loaded onto NuPAGE 4 to 12% Bis-Tris protein gels (Life Technologies). The proteins were transferred using iBlot 2 transfer stacks (Life Technologies), and the membrane was blocked with 5% dried skimmed milk (Marvel). The HA and NP proteins were probed with human anti-H7N9 HA monoclonal antibodies and mouse anti-NP monoclonal antibody (ATCC), respectively, followed by incubation with IRDye680RD goat anti-mouse IgG and IRDye800CW goat anti-human IgG secondary antibodies and visualization using the Odyssey Clx system (Li-Cor).

### Biolayer interferometry.

The influenza virus receptor binding affinity was measured on an Octet Red instrument (FortéBio). The equilibrium responses for virus binding were plotted as a function of the amount of sugar immobilized on the biosensor calculated from the response during the sugar loading step (35). Briefly, influenza viruses were purified by a continuous 30 to 60% (wt/vol) sucrose gradient, and the concentration of viruses was determined using an enzyme-linked immunosorbent assay against the virus NP as described previously (35). The biotinylated α2,3- and α2,6-linked sialyl lactosamine sugars (3SLN and 6SLN, respectively) were purchased from GlycoNZ. Virus was diluted in HEPES-Buffered Saline with EDTA and Polysorbate (Tween 20) (HBS-EP) buffer (Teknova) containing 10 μM oseltamivir carboxylate (Roche) and 10 μM zanamivir (GSK) to a final concentration of 100 pM. Equilibrium responses for virus binding were plotted as a function of the amount of sugar immobilized on the biosensor calculated from the response during the sugar loading step (35).

### Statistical analysis.

Statistical analysis was performed using GraphPad Prism 8 (GraphPad Software). One-way analysis of variance (ANOVA) was used to test differences between different groups for Fig. 3A to C and Fig. 4C and D. Two-way ANOVA (Tukey’s multiple-comparison test) was used for Fig. 4A and B. *P* values of <0.05 were considered significant.
