# Supplementary material for: Antigenic Characterization of Human Monoclonal Antibodies for Therapeutic Use against H7N9 Avian Influenza Virus
Source: J Virol. 2022 Dec 21;97(1):e01431-22. doi: 10.1128/jvi.01431-22 (PMC9888198; doi:10.1128/jvi.01431-22)
Supplement: Supplemental file 1 — Tables S1 and S2. Download jvi.01431-22-s0001.pdf, PDF file, 0.2 MB [file jvi.01431-22-s0001.pdf]

Antigenic characterisation of human monoclonal antibodies for therapeutic use against H7N9 avian influenza virus

Running title: Antigenic mapping of H7N9 AIV

Pengxiang Chang[1], Deimante Lukosaityte[1], Joshua E. Sealy[1], Pramila Rijal[2,3], Jean-Remy Sadeyen[1], Sushant Bhat[1], Sylvia Crossley[1], Rebecca Daines[1], Kuan-Yin A. Huang[4,5], Alain R. Townsend[2,3], Munir Iqbal[1#]

[1]The Pirbright Institute, Pirbright, Woking, GU24 0NF, UK.

[2]Center for translational Immunology, Chinese Academy of Medical Science Oxford Institute, Nuffield Department of Medicine, University of Oxford, Oxford, OX3 9DS, United Kingdom.

[3]MRC Human Immunology Unit, MRC Weatherall Institute of Molecular Medicine, Radcliffe Department of Medicine, University of Oxford, UK.

[4]Graduate Institute of Immunology, College of Medicine, National Taiwan University, Taipei, Taiwan.

[5]Department of Pediatrics, National Taiwan University Hospital, Taipei, Taiwan.

Supplementary table 1:

The two-way ANOVA of the H7N9 avian influenza virus replication kinetics in MDCK cells

Tukey's multiple-comparisons test parameters [a]	Significant	Adjusted *P* value
15 h:Wild-type vs. 15 h:A125T	Yes	<0.0001
15 h:Wild-type vs. 15 h:G133E	Yes	<0.0001
15 h:Wild-type vs. 15 h:N149D	Yes	<0.0001
15 h:Wild-type vs. 15 h:L217Q	Yes	<0.0001
24 h:Wild-type vs. 24 h:A125T	No	0.1827
24 h:Wild-type vs. 24 h:G133E	No	0.9994
24 h:Wild-type vs. 24 h:N149D	Yes	0.003
24 h:Wild-type vs. 24 h:L217Q	No	>0.9999
48 h:Wild-type vs. 48 h:A125T	Yes	<0.0001
48 h:Wild-type vs. 48 h:G133E	Yes	0.0001
48 h:Wild-type vs. 48 h:N149D	Yes	<0.0001
48 h:Wild-type vs. 48 h:L217Q	Yes	0.0495
72 h:Wild-type vs. 72 h:A125T	Yes	<0.0001
72 h:Wild-type vs. 72 h:G133E	Yes	0.0125
72 h:Wild-type vs. 72 h:N149D	Yes	<0.0001
72 h:Wild-type vs. 72 h:L217Q	No	0.9958

[a] Wild-type and mutant viruses with indicated amino acid substitutions were generated via reverse genetic approach contained HA and NA from Anhui/13 virus and the internal gene segments from PR8 virus. MDCK cells were infected with viruses at an MOI of 0.001.

Supplementary table 2:

The two-way ANOVA of the H7N9 virus replication kinetics in SIAT cells

Tukey's multiple-comparisons test parameters [a]	Significant	Adjusted *P* value
15 h:Wild-type vs. 15 h:A125T	No	0.6781
15 h:Wild-type vs. 15 h:G133E	No	0.4411
15 h:Wild-type vs. 15 h:N149D	Yes	0.0006
15 h:Wild-type vs. 15 h:L217Q	No	0.6078
24 h:Wild-type vs. 24 h:A125T	Yes	0.0253
24 h:Wild-type vs. 24 h:G133E	No	0.9738
24 h:Wild-type vs. 24 h:N149D	Yes	0.0019
24 h:Wild-type vs. 24 h:L217Q	No	0.9984
48 h:Wild-type vs. 48 h:A125T	Yes	0.0018
48 h:Wild-type vs. 48 h:G133E	No	0.5189
48 h:Wild-type vs. 48 h:N149D	Yes	<0.0001
48 h:Wild-type vs. 48 h:L217Q	No	>0.9999
72 h:Wild-type vs. 72 h:A125T	Yes	0.0002
72 h:Wild-type vs. 72 h:G133E	No	0.3761
72 h:Wild-type vs. 72 h:N149D	Yes	<0.0001
72 h:Wild-type vs. 72 h:L217Q	No	>0.9999

[a] Wild-type and mutant viruses with indicated amino acid substitutions were generated via reverse genetic approach contained HA and NA from Anhui/13 virus and the internal gene segments from PR8 virus. SIAT cells were infected with viruses at an MOI of 0.001.
